# Atrial Structural Remodeling Gene Variants in Patients with Atrial Fibrillation

**DOI:** 10.1155/2018/4862480

**Published:** 2018-09-10

**Authors:** Rosa Doñate Puertas, Gilles Millat, Isabelle Ernens, Vincent Gache, Samuel Chauveau, Elodie Morel, Emilie Christin, Nathalie Couturier, Yvan Devaux, Philippe Chevalier

**Affiliations:** ^1^Institut NeuroMyoGène (INMG), UMR CNRS 5310-INSERM U1217/University of Lyon, Lyon, France; ^2^Laboratory of Molecular Cardiogenetics, Hospices Civils de Lyon, Lyon, France; ^3^NGS Sequencing Platform for Molecular Diagnosis, Hospices Civils de Lyon, Lyon, France; ^4^Cardiovascular Research Unit, Luxembourg Institute of Health, Luxembourg; ^5^Rhythmology Unit, Louis Pradel Cardiology Hospital, Hospices Civils de Lyon, Lyon, France

## Abstract

Atrial fibrillation (AF) is a common arrhythmia for which the genetic studies mainly focused on the genes involved in electrical remodeling, rather than left atrial muscle remodeling. To identify rare variants involved in atrial myopathy using mutational screening, a high-throughput next-generation sequencing (NGS) workflow was developed based on a custom AmpliSeq™ panel of 55 genes potentially involved in atrial myopathy. This workflow was applied to a cohort of 94 patients with AF, 76 with atrial dilatation and 18 without. Bioinformatic analyses used NextGENe® software and* in silico* tools for variant interpretation. The AmpliSeq custom-made panel efficiently explored 96.58% of the targeted sequences. Based on* in silico* analysis, 11 potentially pathogenic missense variants were identified that were not previously associated with AF. These variants were located in genes involved in atrial tissue structural remodeling. Three patients were also carriers of potential variants in prevalent arrhythmia-causing genes, usually associated with AF. Most of the variants were found in patients with atrial dilatation (n=9, 82%). This NGS approach was a sensitive and specific method that identified 11 potentially pathogenic variants, which are likely to play roles in the predisposition to left atrial myopathy. Functional studies are needed to confirm their pathogenicity.

## 1. Introduction

Atrial fibrillation (AF) is the most frequent arrhythmia, affecting 30 million individuals worldwide [1]. Advanced age and hypertension, which can damage the left atrium (LA), are the main predisposing risk factors for AF [2]. A plethora of evidence suggests that the onset of most AF types is facilitated by LA remodeling, i.e., atrial myopathy [3]. Ion-channel, neural, and structural remodeling of the LA muscle has been widely documented [4] and numerous studies have found a genetic predisposition and a highly heritable component associated with AF risk [5].

In the past 20 years, the genetic basis for AF was established through studies evaluating familial AF [6, 7], linkage [8, 9], candidate genes [10, 11], and genome-wide association studies (GWAS) [12–14] that reported common and rare variants in genes encoding ion-channels, gap junction proteins, and signaling molecules. Recently, next-generation sequencing (NGS) technologies have advanced in terms of sensibility, specificity, practicability, and the cost to rapidly screen large numbers of genes. Massively parallel NGS approaches, including gene panels, whole exome sequencing, or whole genome sequencing, are beginning to supplant Sanger sequencing [15]. Thus, sequencing candidate genes might be the best approach to reveal variations in AF-associated genes [16–18].

The available molecular data only account for a limited percentage of the genes involved in AF, mainly those involved in ion-channel remodeling. Atrial myocardial damage is characterized by atrial fibrosis [19], inflammatory infiltrates [20], altered cell-to-cell adhesion and mechanical coupling [21], and abnormal contractions [22]. To identify variants in the genes coding for proteins potentially involved in atrial tissue rather than ion-channel remodeling, we designed a fast protocol utilizing a custom AmpliSeq panel and Ion Personal Genome Machine (PGM) Sequencer to sequence 55 atrial myopathy candidate genes in a prospective cohort of 94 patients, 76 with and 18 without atrial dilatation. Patients carrying pathogenic or likely pathogenic variants were also screened against a homemade panel of prevalent arrhythmia-causing genes, mainly involved in electrical remodeling.

## 2. Materials and Methods

### 2.1. Patients

The cohort included 94 nonvalvular patients with AF prospectively recruited from the Louis Pradel Cardiology Hospital (Hospices Civils de Lyon, Lyon, France). The ethics committee of Lyon approved the study and informed consent was obtained from each patient prior to enrollment (DC2015-2566). Individuals older than 18 years with a confirmed diagnosis of paroxysmal/persistent/permanent AF but without significant underlying heart disease, left ventricular dysfunction (left ventricular ejection fraction <50%), valvular heart diseases, or other systemic/metabolic diseases were included in the cohort. The presence of AF was determined by ECG or Holter recordings. Paroxysmal AF was defined as self-terminating, in most cases within 48 hours. Persistent AF lasted longer than 7 days and was terminated by either pharmacologic intervention or electrical cardioversion. For permanent AF, rhythm control interventions were not pursued [23]. Left atrial dilatation was defined as a volume superior to 32 ml/m^2^ or a surface > 22 cm^2^, measured by transthoracic echocardiography.

### 2.2. NGS Strategy

Genomic DNA samples underwent NGS using a custom AmpliSeq design (Life Technologies, Carlsbad, CA, USA) created using Ion AmpliSeq designer software. In the first step, the criteria for gene selection were based on the previously reported transcriptome of atrial tissue in patients with AF [24]. We found that 1,627 genes had altered basal expression levels in the LA tissue of patients with AF compared with the control group. The significantly enriched Gene Ontology biological process “anatomical structure morphogenesis” contained the highest number of genes, and this was in line with changes in structure that occur when the human heart remodels following AF development (i.e., left atrial dilatation and interstitial fibrosis). We then selected the most dysregulated genes to build a homemade gene panel. In the second step, genes were selected, using PubMed, based on their documented or potential involvement in structural remodeling. A candidate ID gene list was generated using the search terms: “structural remodeling”, “AF fibrosis”, “AF conduction”, and “AF inflammation”. Articles concerning the structural remodeling of AF were included predominantly in the list. The genetic panel was made of 55 genes potentially involved in structural heart disease (Table 1). The design allowed analysis of all coding exons of selected genes (padding ±30 bp). Library preparation and Ion Torrent PGM sequencing were performed as previously reported [25, 26]. Selected patients carrying pathogenic or likely pathogenic variants in this panel were further tested by NGS using a second custom panel designed to identify disease-causing variants in 38 known arrhythmia-causing genes [27].

### 2.3. Bioinformatic Analyses

**Figure 1:**
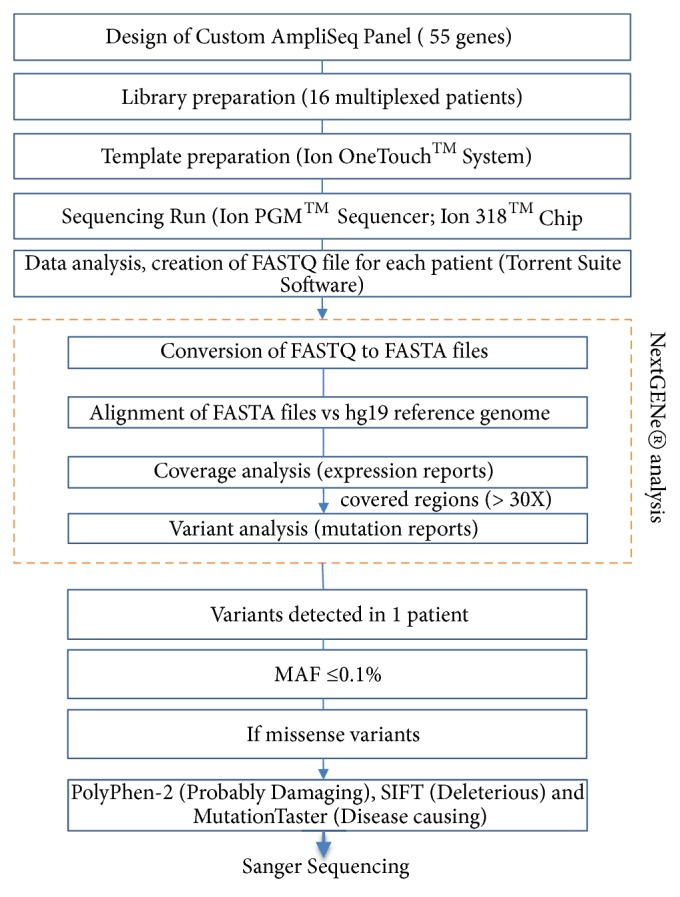
*Filtering steps*. Decision tree for exploration of genes related to atrial fibrillation using a next-generation sequencing approach to detect mutations, based on a custom AmpliSeq library and Ion Torrent PGM sequencing. Abbreviations: MAF = minor allele frequency; PGM = Personal Genome Machine.

Bioinformatic analyses were performed using a homemade pipeline based on Next*GEN*e v.2.3.4.2 (SoftGenetics, State College, PA, USA) and Alamut® 2.7.1 (Interactive Biosoftware, Rouen, France) software, as previously reported [25, 26]. Identified gene variants (i.e., missense, nonsynonymous, splice site, insertions, and deletions) were further analysed using the filtering steps shown in Figure 1. According to reported guidelines, specific standard terms [“pathogenic”, “likely pathogenic”, “uncertain significance”, “likely benign”, and “benign”] were used to evaluate the pathogenicity of variants identified in the studied genes [28]. Likely pathogenic variants (single nucleotide variants) were verified by conventional dideoxy sequencing using the BigDye® Terminator v.3.1 Cycle Sequencing Kit (Life Technologies, Carlsbad, CA, USA) and an ABI 3730 automatic sequencer (Life Technologies).

The frequency of each variant in the general population was examined using the disease database ClinVar (https://www.ncbi.nlm.nih.gov/clinvar/) and the population database Exome Aggregation Consortium (ExAC) (http://exac.broadinstitute.org/). In* silico* tools used for missense variant interpretation included PolyPhen-2 [29], SIFT [30], and MutationTaster [31]. The grade of evolutionary nucleotide conservation was determined by PhyloP scores (http://compgen.cshl.edu/phast/). The protein evolution was predicted with the Grantham score [32]. The protein domains affected by the single nucleotide changes were also described. Multiple protein sequences across species were aligned using the program MUSCLE [33] version 3.6.

### 2.4. Quantification Methods

Nuclear positioning was quantified in mammalian myotubes containing at least five nuclei, and myotubes were classified aggregated when more than 70% of the nuclei did not align along the same axis.

### 2.5. Transfections

Myoblasts were transfected with siRNA using Lipofectamine RNAiMAX (Invitrogen): siRNA sequences (Ambion): 5′-GCUCUAAACAUGAUUCAAGTT-3′ (AKPA9-#1); 5′-CGAUGGUAGAAUUCUUAGATT-3′ (AKPA9-#2); 5′-GCCAAGCUUGUCCAUUGAU-TT-3′ (AKPA9-#3).

### 2.6. Cell Culture

C2C12 myoblasts were grown and differentiated for 5 days as described before [34].

### 2.7. Statistical Analysis

Student's t-tests were performed. Differences were considered statistically significant when P< 0.01.

## 3. Results

**Figure 2:**
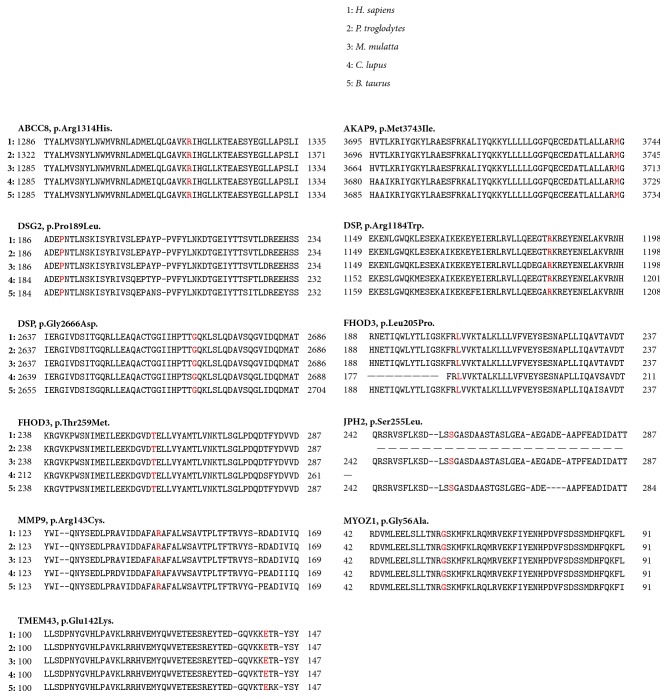
*High conservation across species*. Multiple protein sequence alignments and the evolutionary conservation of each altered amino acid among species (*H. sapiens, P. troglodytes, M. mulatta, C. lupus,* and* B. taurus*).

Clinical features of the 63 men and 31 women included in the cohort are listed in Table 2. The median age at the time of inclusion for AF probands was 54.4 years (range: 42–66 years). Paroxysmal AF was the most common type and 80.8% of patients with AF presented with left atrial dilatation. Particularly, patients developing permanent AF presented left atrial dilatation. Our AmpliSeq custom-made panel explored 96.58% of targeted sequences. Six runs, containing 16 DNA samples each, were performed and the coverage statistics were comparable between each run. The strategy for filtering (Figure 1) led to the identification of 11 putative pathogenic variants not previously reported in patients with AF (Table 3). Each variant was present in a single patient. Nine variants were found in patients with AF and left atrial dilatation and two in patients without atrial myopathy. Three variants were not reported in the ExAC consortium. All putative pathogenic missense variants were predicted to disrupt protein function by PolyPhen-2 (score ranges: 0 to 1), SIFT, and MutationTaster as “probably damaging” (0.85 = the threshold), “deleterious”, and “disease causing”, respectively. The PhyloP highlighted that mutated nucleotides detected in cases 6211, 4464, 2095, 1885, 4162, 1875, and 2691 were highly conserved. Comparisons based on the physical or chemical properties of amino acids showed the candidate* JPH2* variant (p.Ser255Leu) and* MMP9* variant (p.Arg143Cys) had high Grantham differences, suggesting that these missense variants could be pathogenic. A multialignment of proteins (Figure 2) showed that all altered amino acids had high evolutionary conservation across species, suggesting that they could be functionally important.

**Figure 3:**
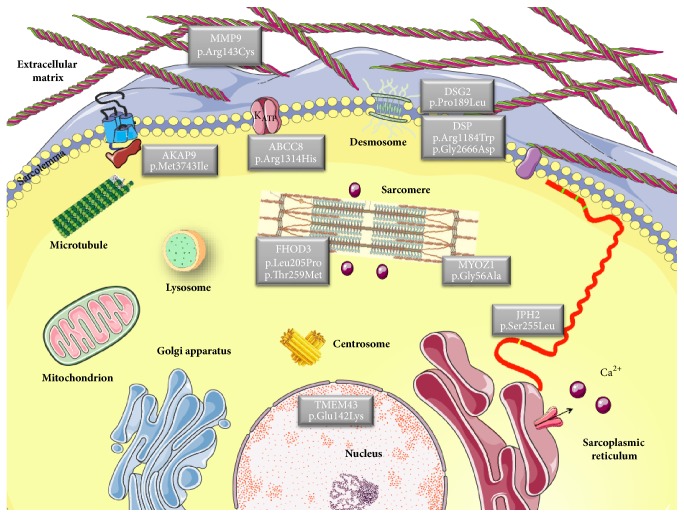
*Atrial fibrillation disease genes.* A schematic of proteins encoded by genes related to atrial fibrillation and their subcellular localization. Proteins participate in many diverse biological processes of cardiomyocytes/fibroblasts.

The 11 patients identified with variants involved in structural remodeling were further screened using an arrhythmia panel with genes known to be associated with AF [27]. Three of these patients were also carriers of likely pathogenic variants in AF-associated genes (Table 4). Left atrial dilatation was also a characteristic of these patients. Only eight patients were carriers of likely pathogenic variants in atrial myopathy genes. An overview of AF-associated genes is displayed in Table 5. The majority of these genes are linked with other cardiac diseases. The cellular localization of proteins encoded by candidate genes is shown in Figure 3.

**Figure 4:**
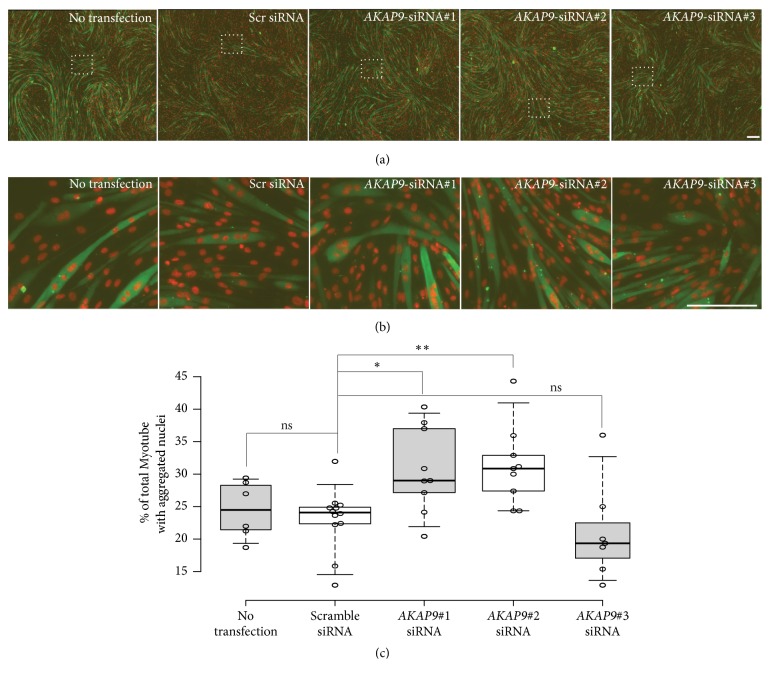
*AKAP9 is required for myonuclear positioning in C2C12 myotubes.* (a) Representative immunofluorescence images of control (no transfection and Scramble siRNA treated cells) or* AKAP9*-depleted C2C12 myotubes (using 3 individual siRNA, 30 nM each) differentiated for 5 days and immunostained for myosin heavy chain (green) and 49,6-diamidino-2-phenylindole (red). Scale bar, 160 um. (b) 7× magnifications of rectangles shown in images (a). Scale bar, 160 um. (c) Histogram of percentage of total C2C12 myotubes with aggregated nuclei control (no transfection and Scramble siRNA treated cells) or* AKAP9*-depleted C2C12 myotubes (using 3 individual siRNA, 30 nM each) differentiated for 5 days. Center lines show the medians; box limits indicate the 25th and 75th percentiles as determined by R software; whiskers extend to 5th and 95th percentiles, outliers are represented by dots; width of the boxes is proportional to the square root of the sample size; data points are plotted as open circles. n = 6, 12, 9, 9, 7 sample points. Student's t-tests were performed between scrambled siRNA and experimental condition. Asterisk, P, 0.05; two asterisks, P, 0.01; ns: nonsignificant.

*AKAP9* encodes a scaffolding protein involved in Golgi apparatus integrity and Golgi-related microtubules nucleation [35]. It has been recently shown that* AKAP9* can contribute to recruit microtubule-organizing center factors at the membrane of myonuclei [36]. We validated* AKAP9*-dependent myonuclei positioning in a muscle cells context using C2C12 myoblast and quantify myonuclei aggregation in* AKAP9*-depleted myotubes using 3 different siRNA (Figure 4).* AKAP9*-depleted myotubes significantly increase myonuclei aggregation phenotype (up to 30%) within myotubes (Figure 4(c)) without affecting myoblast fusion or myotubes differentiation (Figures 4(a) and 4(b)), confirming a microtubule integrity regulation by an* AKAP9*-dependant mechanism in a muscle cells context [36].

## 4. Discussion

This study identified 11 potentially pathogenic variants in patients with AF, using a simple and fast NGS mutation detection approach. In contrast with previous studies, our method focused on the identification of candidate gene variants not previously linked to AF-structural remodeling genes. The role of genetic factors in the development of AF, a complex and multifactorial arrhythmia, is increasingly recognized. At least 14 genetic loci revealed by GWAS are known to increase the risk of AF in populations [37], but these variants only explain a small fraction of the interindividual risk for AF. Most identified genetic loci are associated with genes of electrical remodeling, such as* KCNN3* [13], or developmental genes, such as* PITX2* [12]. However, a meta-analysis of GWAS suggested additional candidate AF loci, such as genes involved in structural components (*SYNE2*,* MYOZ1*, and* SYNPO2L)* [14]. The NGS represents a high-throughput, rapid, and low-cost strategy for the systematic detection of genomic variants involved in AF. Our NGS approach was based on a custom AmpliSeq design to detect variants in structural remodeling genes. The filtering strategy allowed us to identify 11 rare variants. For all variants,* in silico* tools were used to predict the possible pathogenic impact of an amino acid substitution on the structure and function of the human proteins. This predicted deleterious impact of these variants was strengthened by the evolutionary conservation of the altered amino acids.

Our initial hypothesis was that structural genes could be involved in atrial remodeling as much as ion-channel ones. Three likely pathogenic variants were in ion-channel genes previously associated with AF. Defects were found in* ANK2*, which encodes a multifunctional cytoskeletal adaptor [38],* KCNH2*, which encodes a potassium voltage-gated channel, and* SCN1B*, which encodes the *β*-subunit of the sodium channel [39]. Evaluation of the missense variants using both segregation data and* in vitro* systems may help better understand the pathogenicity. The substitution at the splice donor site of the* SCN1B* intron 1, which was not reported in the ExAC consortium, is expected to yield a nonsense-mediated decay mechanism, resulting in a reduction of protein and haploinsufficiency. Several studies have shown that atrial dilatation is an independent risk factor for the development of AF [40]. In a recent study of eight patients with AF and a frameshift deletion in* MYL4*, six subjects developed LA dilatation during the follow-up [22]. In the present study, 82% of the novel variants were found in patients with LA dilatation, reinforcing the suggestion that these variants could be involved in LA structural damage. Most of the identified genes were previously linked to other cardiac diseases (Table 5).* AKAP9, FHOD3*, and* TMEM43* were not previously associated with AF in the literature, but they were linked with other cardiac diseases.

The majority of the new variants found in the present study are located in genes encoding a broad category of proteins. These proteins are involved in many diverse biological processes related to structural remodeling of the extracellular matrix, the sarcolemma, the cytoskeleton, desmosome, sarcomere, the sarcoplasmic reticulum, and nucleus. Upregulation of* MMP9*, a profibrotic and proinflammatory molecule, contributes to atrial extracellular matrix remodeling [41], which is associated with the development of AF [42]. In the sarcolemmal ATP-sensitive potassium channels of the cardiomyocytes,* ABCC8* encodes the regulatory sulfonylurea receptor 1. Proteins involved in the desmosome structure include that encoded by* DSG2* and* DSP*.* DSG2* is more expressed in LA of patients with AF than control subjects as previously described [24]. Transcriptional network of cardiac rhythm driven by* TBX5* and modulated by* PITX2* regulates* Scn5a*,* Gja1*,* Ryr2*,* Dsp*, and* Atp2a2* genes [43]. Some of the proteins associated with the selected variants contribute to the structure or function of the sarcomere, with* FHOD3* playing a role in regulation of the actin filament assembly [44]. The cell structure gene* MYOZ1* encodes myozenin-1, which is a skeletal muscle Z line protein involved in stabilizing the sarcomere [45]. In addition,* JPH2* encodes a cardiac structural protein contributing to the formation of the junctional membrane complex architecture that links the sarcoplasmic reticulum with the plasma membrane in cardiomyocytes [46]. The* JPH2* mutation is thought to cause AF because of impaired stabilization of ryanodine receptor Ca2+ channels [47]. The inner nuclear membrane contains associated proteins, including that encoded by* TMEM43*, which is associated with lamin A/C and emerin [48].* AKAP9*, a scaffolding protein involved in Golgi apparatus integrity and Golgi-related microtubules nucleation [35], is known to be the long QT syndrome-causative gene [49]. Our results confirmed an altered microtubule network in absence of* AKAP9* as inhibition of* AKAP9* results in increased aggregation phenotype in myotubes [36]. Consequences of* AKAP9* knockdown on remaining pool of microtubule-associate-partners remain to be determines. One can speculate that forces exerted by muscle molecular motors could be remodel in absence (or mutated forms) of* AKAP9* and could contribute to alteration of microtubule network dynamic [50, 51]. Microtubules networks are mechanically involved in cardiomyocyte contraction [52]. It will be of interest to analyse resulting network depending on different* AKAP9* variant and skeletal muscle cells could be used as a *«*simplified muscle model*»* to screen for the effect on microtubule dynamics of different variant of* AKAP9* found in cardiac muscles.

Each of these variants is involved in different pathways. The link between these variants and the effect on gene expression is unclear. A recent study has found that the SNP rs2595104 associated with AF regulates PITX2c expression via interaction with TFAP2a [53]. MiRNAs are part of the molecular alterations in LA occurring in patients with atrial remodeling [54]. One might consider that a variant could regulate miRNA in AF patients [55]. Cumulative evidence suggests that response to therapy may be genotype dependent. For example, SNP on chromosome 4q25 associated with AF modulates response to antiarrhythmic therapy [56]. This work opens research directions to establish personalised therapies according to individual genomic data as in cancer patients [57].

## 5. Conclusions 

Eleven rare or novel potentially pathogenic variants were identified using the NGS method in patients with nonvalvular AF, mainly in those with atrial dilatation. Validation studies are needed to confirm the involvement of these variants in atrial structural remodeling. This approach (Figure S1), based on genes involved in atrial structural remodeling, may help uncover new mechanisms underlying AF. In addition, candidate gene approaches based on disease physiopathology should be encouraged.

## Figures and Tables

**Table 1:** List of the genes included in our panel.

**Gene**	**NM number**	**Gene**	**NM number**	**Gene**	**NM number**	**Gene**	**NM number**	**Gene**	**NM number**
*ABCC8*	NM_001287174.1	*FHOD3*	NM_025135.4	*IL10*	NM_000572.2	*MYL7*	NM_021223.2	*SMAD2*	NM_005901.5
*ABCC9*	NM_005691.3	*FKRP*	NM_001039885.2	*JPH2*	NM_020433.4	*MYOZ1*	NM_021245.3	*SMAD3*	NM_005902.3
*ACE*	NM_000789.3	*GATA4*	NM_001308093.1	*JUP*	NM_002230.2	*NODAL*	NM_018055.4	*SMAD4*	NM_005359.5
*AKAP9*	NM_005751.4	*GATA5*	NM_080473.4	*LEFTY1*	NM_020997.3	*NOS1AP*	NM_014697.2	*SMYD2*	NM_020197.2
*CER1*	NM_005454.2	*GATA6*	NM_005257.5	*LEFTY2*	NM_003240.3	*NOS3*	NM_000603.4	*TGFB1 *	NM_000660.5
*CTGF*	NM_001901.2	*GJA1*	NM_000165.4	*LMNA*	NM_170707.3	*ORAI1*	NM_032790.3	*TGFB3*	NM_003239.3
*DES*	NM_001927.3	*GJA5*	NM_005266.6	*LTB2*	NM_002341.1	*PCSK6 *	NM_002570.3	*TIMP-1*	NM_003254.2
*DSC2*	NM_024422.4	*GJC1*	NM_001080383.1	*MMP2*	NM_004530.5	*PITX2*	NM_153427.2	*TIMP-2*	NM_003255.4
*DSG2*	NM_001943.4	*GNB3*	NM_002075.3	*MMP9*	NM_004994.2	*PKP2*	NM_001005242.2	*TMEM43*	NM_024334.2
*DSP*	NM_004415.3	*HSP90AB1*	NM_001271971.1	*MYBPC2*	NM_004533.3	*PRRX1*	NM_006902.4	*TMPO*	NM_003276.2
*FGFR1*	NM_023110.2	*HSPE1 *	NM_002157.2	*MYH7*	NM_000257.3	*SHOX2*	NM_003030.4	*TRPM4*	NM_017636.3

**Table 2:** Clinical parameters of the patients involved in the study.

	All patients (n=94)
Ratio M/F	63/31
Age (years)	54.4 ( ± 12.0)
Age of AF onset (years)	48.01 ( ± 14.35)
BMI	27.7 ( ± 5.5)
* AF type*	
Paroxysmal	55
Persistent	21
Permanent	18
* Risk factors*	
Hypertension	23
CVA	16
LA surface (cm^2^)	26.8 ( ± 7.5)

*Abbreviations*. AF = atrial fibrillation; BMI= body mass index; CVA = cerebrovascular accident; LA = left atrium.

**Table 3:** List of putative pathogenic variations identified in a cohort 94 patients.

**Case**	**Patient characteristics**		**Variation**						**Presence in databases**		**Nucleotide conservation prediction**	**Grantham Score** ^¶^	**Protein domain**
	**AF type**	**Dilated LA** ^†^	**Gene**	**Chr**	**Exon**	**Nucleotide change**	**Effect on protein**	**Pathogenicity** ^‡^	**ClinVar**	**ExAC**	**PhyloP** ^§^		

2115	*Paroxysmal*	*Yes*	*ABCC8*	11	32	c.3941G>A	p.Arg1314His	Likely pathogenic		Yes	3.98	29	ABC transporter type 1, transmembrane domain

6211	*Permanent*	*Yes*	*AKAP9*	7	46	c.11229G>A	p.Met3743Ile	Likely pathogenic	RCV000310251.1 (Uncertain significance), RCV000171732.1 (Likely benign), RCV000362669.1 (Uncertain significance)	Yes	7.99	10	Pericentrin/AKAP-450 centrosomal targeting domain

6198	*Permanent*	*Yes*	*DSG2*	18	6	c.566C>T	p.Pro189Leu	Likely pathogenic		No	1.50	98	Cadherin; Cadherin-like

2235	*Paroxysmal*	*No*	*DSP*	6	23	c.3550C>T	p.Arg1184Trp	Likely pathogenic		Yes	3.98	101	

4464	*Permanent*	*Yes*	*DSP*	6	24	c.7997G>A	p.Gly2666Asp	Likely pathogenic		Yes	10.00	94	Plectin repeat

2095	*Paroxysmal*	*Yes*	*FHOD3*	18	7	c.614T>C	p.Leu205Pro	Likely pathogenic		No	7.99	98	Rho GTPase-binding/formin homology 3 (GBD/FH3) domain; Armadillo-type fold

1885	*Paroxysmal*	*Yes*	*FHOD3*	18	8	c.776C>T	p.Thr259Met	Likely pathogenic		Yes	7.76	81	Rho GTPase-binding/formin homology 3 (GBD/FH3) domain; Armadillo-type fold

4162	*Permanent*	*Yes*	*JPH2*	20	2	c.764C>T	p.Ser255Leu	Likely pathogenic		No	9.18	145	Junctophilin

2186	*Paroxysmal*	*Yes*	*MMP9*	20	3	c.427C>T	p.Arg143Cys	Likely pathogenic		Yes	2.68	180	Peptidase M10, metallopeptidase; Peptidase, metallopeptidase; Peptidase M10A

1875	*Paroxysmal*	*No*	*MYOZ1*	10	3	c.167G>C	p.Gly56Ala	Likely pathogenic		Yes	7.76	60	Myozenin

2691	*Paroxysmal*	*Yes*	*TMEM43*	3	5	c.424G>A	p.Glu142Lys	Likely pathogenic	RCV000039386.3 (Uncertain significance), RCV000172593.3 (Likely benign), RCV000250239.1 (Uncertain significance)	Yes	8.11	56	Transmembrane protein 43 family

^†^ LA volume >32 ml/m^2^ or a surface >22 cm^2^; ^‡^specific standard terminologies—“pathogenic”, “likely pathogenic”, “uncertain significance”, “likely benign”, and “benign” were used to describe variants identified (Ref [28]); ^§^ range of PhyloP score [-20.0;10.0]; ^¶^ range of Grantham score [0-215]. Abbreviations: AF = atrial fibrillation; ExAC = Exome Aggregation Consortium; LA= left atrium.

**Table 4:** List of variants identified in the prevalent arrhythmia-causing genes.

**Case**	**Variation **							**Presence in databases**		**Prediction analysis of missense variants**			**Nucleotide conservation prediction**	**Grantham Score** ^§^
	**Gene **	**NM_number **	**Chr**	**Exon/ intron **	**Nucleotide change**	**Effect on protein**	**Pathogenicity ** ^†^	**ClinVar**	**ExAC**	**PolyPhen-2**	**SIFT**	**Mutation Taster**	**PhyloP ** ^‡^	
2095	*ANK2*	NM_001148.4	4	35	c.4315G>A	p.Gly1439Ser	Likely pathogenic		No	Possibly damaging	Tolerated	Disease causing	2.80	56
6198	*KCNH2*	NM_000238.3	7	11	c.2681G>A	p.Arg894His	Likely pathogenic		Yes (MAF ≤0.01%)	Probably damaging	Tolerated	Disease causing	2.68	29
1885	*KCNH2*	NM_000238.4	7	13	c.3052C>G	p.Pro1018Ala	Uncertain significance	RCV000181908.1 (Uncertain significance)	Yes (MAF ≤0.01%)	Benign	Tolerated	Disease causing	1.50	27
1885	*SCN1B*	NM_001037.4	19	intron 1	c.40+2T>G		Likely pathogenic		No					

^†^ Specific standard terminologies—“pathogenic”, “likely pathogenic”, “uncertain significance”, “likely benign” and “benign” were used to describe variants identified [28]; ^‡^ range of PhyloP score [-20.0;10.0]; ^§^ range of Grantham score [0-215]. Abbreviations: ExAC = Exome Aggregation Consortium; MAF = minor allele frequency.

**Table 5:** Genes associated with cardiac diseases.

**Gene**	**Protein**	**Cardiac diseases classification**							**Protein localisation**
		**LQTS**	**SQTS**	**BrS**	**SIDS**	**CDM**	**CHD**	**AF**	
*ABCC8*	Sulfonylurea receptor 1	X	–	–	–	X	X	X	Sarcolemma
*AKAP9 *	A-kinase anchor protein 9	X	–	X	X	X	–	–	Centrosome
*DSG2*	Desmoglein 2	–	–	X	–	X	–	X	Desmosome
*DSP*	Desmoplakin	X	–	X	X	X	–	X	Desmosome
*FHOD3*	FH1/FH2 domain-containing protein 3	–	–	–	–	X	–	–	Z-disk
*JPH2*	Junctophilin 2	–	–	X	–	X	–	X	Sarcoplasmic reticulum
*MMP9 *	Matrix metalloproteinase-9	X	–	–	–	X	X	X	Extracellular matrix
*MYOZ1*	Myozenin-1	–	–	–	–	X	–	X	Z-disk
*TMEM43*	Transmembrane protein 43	–	–	X	–	X	–	–	Transmembrane

The presence of an (X) indicates involvement of the gene in each specific cardiac disease classification. The localisation of the protein encoded by each disease gene is also described. Abbreviations: BrS = Brugada syndrome; CDM = cardiomyopathies; CHD = congenital heart diseases; LQTS = long QT syndrome; SIDS = sudden infant death syndrome; SQTS = short QT syndrome.

## Data Availability

The sequencing data used to support the findings of this study are available from the corresponding author upon request.

## List of Abbreviations

AF: Atrial fibrillation
ExAC: Exome aggregation consortium
GWAS: Genome-wide association studies
LA: Left atrium
NGS: Next-generation sequencing
PGM: Personal Genome Machine.

## Supplementary Materials

Supplementary Materials Supplementary Figure 1: schematic overview of our approach in atrial fibrillation (AF) patients. According to our previous transcriptomic data on left atrium (LA) in AF patients that highlighted the structural genes [24], a next-generation sequencing (NGS) approach has been applied to a cohort of 94 AF patients. Eleven potentially pathogenic variants were identified in AF patients, mainly in those with atrial dilatation, through a custom-made panel of 55 genes potentially involved in atrial myopathy. These variants were located in genes involved in atrial tissue structural remodeling.
