# Supplementary material for: Atrial Structural Remodeling Gene Variants in Patients with Atrial Fibrillation
Source: Biomed Res Int. 2018 Sep 10;2018:4862480. doi: 10.1155/2018/4862480 (PMC6151856; doi:10.1155/2018/4862480)

Atrial Fibrillation patients

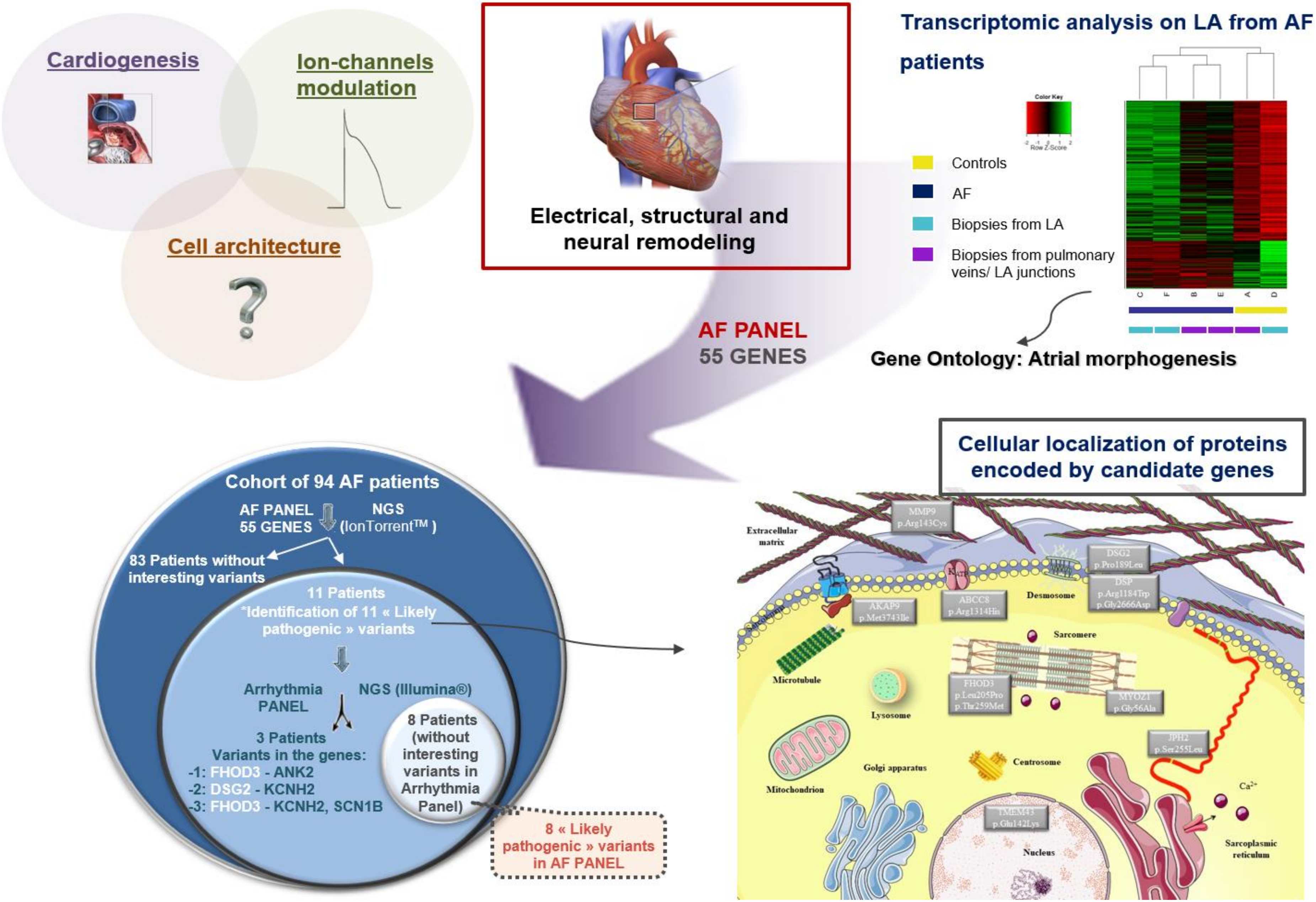

Supplement: Supplementary Materials — Supplementary Figure 1: schematic overview of our approach in atrial fibrillation (AF) patients. According to our previous transcriptomic data on left atrium (LA) in AF patients that highlighted the structural genes [24], a next-generation sequencing (NGS) approach has been applied to a cohort of 94 AF patients. Eleven potentially pathogenic variants were identified in AF patients, mainly in those with atrial dilatation, through a custom-made panel of 55 genes potentially involved in atrial myopathy. These variants were located in genes involved in atrial tissue structural remodeling. [file 4862480.f1.pdf]
